# Initiating Change of People With Criminal Justice Involvement Through Participation in a Drama Project: An Exploratory Study

**DOI:** 10.3389/fpsyt.2019.00716

**Published:** 2019-10-03

**Authors:** Adrian P. Mundt, Pamela Marín, Caroline Gabrysch, Carolina Sepúlveda, Jacqueline Roumeau, Paul Heritage

**Affiliations:** ^1^Medical Faculty, Universidad San Sebastián, Puerto Montt, Chile; ^2^Medical Faculty, Universidad Diego Portales, Santiago, Chile; ^3^Department of Psychiatry and Psychotherapy, Charité Campus Mitte, Universitätsmedizin Berlin, Berlin, Germany; ^4^Department of Psychiatry and Mental Health, Hospital Clínico Universidad de Chile, Santiago, Chile; ^5^Department of Psychology, Universidad del Humanismo Cristiano, Santiago, Chile; ^6^Corporación de Artistas por la Rehabilitación y Reinserción Social a través del Arte CoArtRe, Santiago, Chile; ^7^Department of Drama, Queen Mary University of London, London, United Kingdom

**Keywords:** drama, art therapy, prison population, mental disorders, addiction, substance use disorders

## Abstract

**Introduction:**

Innovative and interdisciplinary approaches are needed to improve mental health and psychosocial outcomes of people with criminal justice involvement and their families. Aim of the study was to assess effects of the participation in a theatre project on the mental health problems of people with criminal justice involvement and relatives.

**Methods:**

We conducted structured diagnostic interviews and in-depth qualitative interviews with five participants performing Shakespeare’s *Richard III* in Chile. Three participants had been imprisoned prior to the project, and two were the parents of a person who died in a prison fire. Qualitative interviews followed a topic guide. Data were transcribed, and a six-phase approach for thematic analysis of the data was used.

**Results:**

Substance use disorder or major depression was identified in all the participants. Participation in the theatre project was experienced by the respondents as having a positive effect on the mental health conditions. The research registered the positive experiences of role identification, emotional expression, commitment with group processes, improved skills to socially interact, to be heard by the general public and society, and positive perceptions of the audience (including relatives).

**Discussion:**

The study raises the possibility that there may be improvements of depression and substance use problems through the participation of people with criminal justice involvement in a drama project. Wider scale research is recommended on the possible effects. The approach may be an alternative to psychotherapy and medication for some individuals.

## Introduction

Prison populations have grown worldwide in the past 15 years (1). In South America, the increase of prison population rates has been especially pronounced (1) and related to a decrease in psychiatric bed numbers (2). There has been concern about high rates of mental health problems (3, 4) and substance use disorders (5, 6) in prison populations (7). Improvements of mental health care and psychosocial outcomes of prison populations are considered a global public health challenge. Innovative and interdisciplinary approaches are needed.

Therapeutic effects of theatre have been described since Aristotle first outlined his theory of catharsis (8–12). Specific drama-based therapeutic approaches based on performance include Moreno’s “Psychodrama,” Augusto Boal’s “Theatre of the Oppressed,” and “Rainbow of Desire” and drama therapy (13–16). Psychodrama allows participants to enact scenes from the past, present or future, and imagined or real, as if they were occurring at the moment in a safe therapeutic environment (15). Boal’s experiments aimed to overcome oppression initiating political change and overthrowing internalized oppression through a dialectic process involving “spect-actors” (13, 14). Drama therapy encourage activities that create a dramatic reality through engagement with embodiment, projection, and roleplay (12). In all the above methodologies, drama is seen to address psychological and social needs such as self-expression, identity, freedom (of imagination), creativity, and community (17).

Art as a vehicle to change behavioral patterns of those making it has been increasingly used in correctional institutions. In qualitative case studies as well as in quantitative evaluations of art projects, it has been shown that art can engage prisoners in transforming aspects of their lives (17, 18). Positive effects on the personalities of imprisoned people and cost-benefits for societies have been described (19, 20, 17, 18). Lower rates of recidivism, the development of coping strategies, and social responsibility as well as the decrease of anger levels have been identified as a result of art programs in prisons (21–24). The unlocking of creative potentials in prisons has been described to have potential for the long-term rehabilitation of participants in music programs (25). Drama-based therapeutic techniques may be more effective than verbal therapies in evoking emotional states and allow more healthy self-reflection in forensic clients with personality disorders (26). Participating in drama seems to improve the skills of people to maintain positive relationships with others. Participants may establish trust and respectful relationships within the group and mutual commitments to a common task. There may be a possibility to challenge negative narrative identities of people with criminal justice involvement. The sense of usefulness and belonging could help in the formation of a pro-social identity (27).

Testimony therapy is a psychotherapeutic method that originated in South America (28). This psychotherapeutic technique of narrating and writing testimonies was used to integrate traumatic experiences of former prisoners, survivors of the military dictatorship in Chile. Narrative exposure therapy is one of the most established trauma-focused psychotherapies, and it was derived from this approach (29, 30). Expression through acting in drama could help to integrate traumatic experiences of people with experience of incarceration and their families (4).

There is further need to understand processes and mechanisms of change related to the participation of people with experience of incarceration and their families in drama projects. The aim of this exploratory study was to assess subjective effects of participating in a drama project on mental health problems and on interpersonal and social functioning in former prisoners. The study also included an assessment of people who have been involved tangentially with the penal system through including the parents of a prisoner who died in a prison fire.

## Methods

### The Theatre Production

CoArtRe (Corporación de Artistas por la Rehabilitación y Reinserción Social a través del Arte) is a nonprofit organization founded in 1998 that realizes theatre projects in criminal justice contexts in Chile. The organization has artistic and social aims (31). Those include translating testimonies of incarcerated individuals in theatre productions and promoting social inclusion and mobility through artistic experiences. Former prison inmates, the parents of a prisoner who had died in prison and professional actors, participated in the production of *Richard III* by Shakespeare. Rehearsals took place four times a week between November 2015 and March 2016. The production premiered in March 2016 in Santiago de Chile (Teatro, Centro Cultural Gabriela Mistral, Chile).

### Shakespeare and Imprisonment

“To hold as ‘twere the mirror up to nature: to show virtue her feature, scorn her own image, and the very age and body of the time his form and pressure” (32). Shakespeare gave these words to Hamlet as he instructed the actors who were taking part in his experimental performance to “catch the conscience” of the King. The London Shakespeare Workout describes Shakespeare’s language as offering an opportunity to change by providing “confidence through the will to dream” (33). The roles in Shakespeare’s plays offer possibilities of identification for people with criminal justice involvement and those affected by imprisonment. Shakespeare’s work is comprised of topics such as crime, factional allegiances, violence, revenge, love, desire, and hatred. His complex characters and their actions have proved themselves resilient across four centuries and continue to connect to different cultures, contexts, and lives today. While the historical distance of these texts and their characters can defuse the issues, questions, and uncertainties they raise, new contexts in which they are expressed (e.g., in relation to the experience of incarceration) can bring new effects, affects, and implications. Working with Shakespeare’s texts can help people get affected by imprisonment to reflect personal choices and to think critically (34, 35). Shakespeare’s work has a special place in prison theatre (36, 37). Combining marginalized prison contexts and allowing performers to bring their biographies and personalities to classical dramaturgic texts through the performance of Shakespeare brings a particular experience of authenticity for the spectators (38).

### *Richard III* by Shakespeare

*Richard III* (c.1593) is one of Shakespeare’s most intriguing works. The protagonist has a complicated character. His sinister character depicts cruelty, while his hunger for power acts as the driving force (39). Richard’s seductive yet ruthless manipulations combine with criminal acts of extreme violence to secure the throne for himself, without respect for family, social norms, or the limits of the law. Yet, at the end of the play, Shakespeare gives us a glimpse of human vulnerability within all the extravagant monstrosities as Richard is haunted by his victims’ spirits, his own nightmares, and an emerging, fearful conscience:

*My conscience hath a thousand several tongues*,

*And every tongue brings in a several tale*,

*And every tale condemns me for a villain* (40)

Perhaps we might say that Richard is a prisoner of his own desires and becomes a victim of the web of intrigues and crimes he himself weaves.

### Sample and Procedures

In order to cast his production *of Richard III*, JR recruited professional actors and participants with different levels of experience of the Chilean penal system. The five non-professional participants had previously participated in a documentary prison theatre production by CoArtRe that focused on a prison fire which took place when three of them were still held in the penal system of Santiago de Chile. By the time of casting for *Richard III*, those three participants had been released and were in the process of psychosocial rehabilitation. They had large roles including the title role. The other two were family members of a prison inmate who had died in the prison fire. Furthermore, professional actors were recruited to perform the remaining roles and included in the crew at later stages of the rehearsals.

Participants were approached for interviews during rehearsal. Qualitative interviews were held in private areas upon written informed consent of the participants. Data were audiotaped and transcribed eliminating information that would allow personal identification. The data were collected between March and June 2016. The research ethics review board of the Universidad San Sebastián, Chile, approved the study.

We conducted structured diagnostic interviews and qualitative interviews with five participants of the theatre project. The interviews were held during the rehearsals and after performing in the play. In total, eight in-depth interviews were audio-recorded (five during rehearsals before the performances, three after performances of the play), subsequently transcribed, and included in the analysis.

### Instruments

The Mini-International Neuropsychiatric Interview (MINI) in Spanish was used to assess mental disorders and substance use disorders. The MINI is a fully structured diagnostic interview (41) that has been used in epidemiological research to establish the prevalence of mental disorders in prison populations in Chile (42). It classifies mental disorders according to the Diagnostic Statistical Manual of Mental Disorders (DSM-IV) and the International Classification of Diseases (ICD-10). Qualitative interviews were guided through topics relating to *expectations with respect to the participation* and *experiences of the participation*. Particular areas covered included: positive/negative effects, social interactions (with other participants), emotions (during participation), perception of rehabilitation (effects of participating), work (effect on personal development), family, and mental health (possible changes in health and mental health).

### Analyses

Mental health and substance use disorders of the participants were described.

Qualitative data were analyzed by CS and CG using an approach consisting of six phases: familiarization, initial coding, searching for themes, reviewing themes, definition of themes, and reporting (43). Interviews were analyzed using an inductive thematic analysis procedure (44, 45). Coding was conducted manually by two researchers CG and CS. Disagreements were resolved in discussion with a third researcher APM. NVivo software was used for the initial quantitative content analysis identifying the most used words.

## Results

### Characteristics of the Sample

Participants were between 36 and 65 years old. Participants with direct experience of criminal justice involvement had served sentences for violent crimes. The structured clinical interviews of the five participants revealed one participant with current illicit drug dependence (former inmate) and three with major depression (one former inmate and the parents of an inmate who died in a prison fire). All had mild suicide risk related to previous suicide attempts (**Table 1**).

**Table 1:** Sociodemographic, criminal, and mental health characteristics of the participants.

Participant identification	TP1	TP2	TP3	TP4	TP5
**Sociodemographic data**					
Sex	m	m	m	f	m
Age	36	44	65	52	36
Marital status	Single	Single	Single	Single	Single
Number of children	3	0	4	3	3
Occupation	Working for income	Working for income	Working for income	Working for income	Working for income
Educational level ISCED	2	1	1	2	2
**Prison related data**					
Criminal justice involvement	Previously imprisoned	Previously imprisoned	Parent	Parent	Previously imprisoned
Offense associated with the longest sentence	Violent crime	Violent crime	x	x	Violent crime
Number of imprisonments	4	1	0	0	2
**Mental health data**					
MINI	Mild suicide risk Hypomania drug and alcohol dependence (past 12 months)	Major depression Mild suicide risk	Major depression Mild suicide risk	Major depression Mild suicide risk	Mild suicide risk

### Coding of the Qualitative Data

The most used words in the interviews served as a first orientation prior to manual coding. The five most used words were *theatre* (*n* = 181), *play* (*n* = 99), *people* (*n* = 55), *prisoner* (Spanish: preso) (*n* = 54), appearing in all eight interviews, and *prison* (Spanish: carcel) (*n* = 45) appearing in six interviews. Inductive analysis resulted in 46 initial codes, which were grouped into 17 categories and two main themes and are reported in **Table 2**.

**Table 2:** Coding, categories, and themes.

Theme (subtheme)	Category	Code	Quote
Type of change and development (previous context)	Personal characteristics	Resources	• TP1 I have always been autodidactic. • TP2 I have capacities to write. • TP4 I am old, but I still continue to learn every day.
		Barriers (former prisoners)	• TP1 I felt ashamed of many things. • TP1 I have difficulties to finish things,…, never or rarely I put things into practice. • TP5 For example … I am very … was very impulsive,…, wherever I arrived, I immediately got into fights.
	Mental health problems	Addictions (former prisoners)	• TP2 I was doing cocaine for 5 years every day. • TP1 I was in a very dark place in this period of drugs and alcohol.
		Depression (parents)	• TP3 I am learning to live with this pain. • TP3 Five years have passed (since the traumatic event), and it still hurts so much (cries). • TP4 I have to go on because there are children suffering, the same as my son suffered.
	Personal reflections	On the own life	• TP1 What was done, is done, what was lost, is lost. If it cannot be recovered, something else can be recovered. • TP1 I did not want this for me, I am already 36 years old, and I have nothing, I do not have any house, I do not have family, my mum is dying, and I will be alone. • TP2 Sadness … I had money, I had a home, I had a wife, I had everything to be happy, and I did not make it … because of my vices (refers to substance use). • TP4 We just have to go forward, nothing else.
		On previous treatments	• TP1 I don’t like therapies, and I don’t like pills. • TP4 And there, I understood that I did not have to use medication, there were other ways.
	Interpersonal relationships	Resources	• TP1 I still have one or other friend. • TP5 I get a lot of support from my family … It consists of my little daughter … and my wife who also makes theatre; she is also an actress; so I have all their support.
		Barriers	• TP1 I abandoned all of my children and did not raise any of them. • TP1 I have a partner who has the same problem, you know? And we are about to break up, despite of all the love, all the tenderness we had because we potentiate each other when it comes to drugs. • TP4 We also had problems with my other son who holds on to me, to not let him alone. He did not want to participate in anything. He was so much affected by the death of his brother, so that we have to care for him now.
	Prison experiences	Repression and poor conditions	• TP5 You had to secretly read. I secretly read books on anarchism (refers to imprisonment). • TP2 Your skin is itching from the bedbugs, and the health is unbearable. In a space,…, a little smaller than this room (about 2 x 3 meters), and it was like 1,80 or 1,90 meters by 2,10 meters were living 8 to 12 people. In a second floor, inserted there were 3, 5, or 10 more…
		Opportunities	• TP1 When I was imprisoned, I was dedicated to learn things, I read, I was informed, I got acquainted with culture, I liked that, I read many books, and I had time. • TP5 For me, the time was not lost. In fact, you could say that, thanks to the imprisonment, I could study drama in the university. • TP2 I armed a rehabilitation center in prison.
		Primera experience with drama	• TP2 And I got hold of a book on drama, and I tried it. • TP5 And then I had this role in the play and started to get into drama.
	Motivation to participate in drama	Previous interest in drama and/or arts (former prisoners)	• TP2 And I continued to make theatre and here I am. • TP5 And then I had this role in the play and started to get into drama. • TP1 I changed to a more quiet cell with other roommates and I still got up, played music, and pointed.
		Need for rehabilitation (former prisoners)	• TP5 And then comes a person and makes plays and gets you out of this and puts you in this world where you start to read texts; you start to put you in a role and you get out of the hangover. • TP2 I had a rehabilitation center in prison.
		Death of a son (parents)	• TP4 Through the death of my son, I got to know theatre. • TP3 As the fortunate consequence of the unfortunate death of my son, we could discover facets and talents of which we did not know that we had…
		Looking for repair (parents)	• TP4 With Torre 5 (title of the play on the prison fire), I discovered everything. I found the whole world except for justice. So you cannot do anything about this, cannot go beyond this. • TP5 This disturbs me. Justice has never come.
		Experience in previous drama production (parents)	• TP3 After a couple of years, she called and I immediately said yes. There was a casting, and I was selected. • TP4 I like it a lot because I learned with the other play we made.
		Expectations to participate in the production	• TP1 I did not dare to speak in public, I got nervous. I think this had to change. • TP1 To acquire more experience with acting to continue participating in the same field. • TP1 (about finding work in the same field) Yes, I think this would be possible. • TP2 If there were possibilities, fantastic. I would like to continue with little things (in the field of theatre). • TP3 Another experience of talent to act and knowing professional actors. • TP5 Indeed I thought about projecting myself in the theatre field, would be great, but there is little money in it, you make very little money.
Type of change and development (perceived changes)	Personal level	Rehabilitation on the personal level (former prisoners)	• TP1 Theatre helped me to engage with people, and in being more disciplined. • TP5 I am, was very impulsive, and the drama has helped me in the personal development … wherever I arrived, I started to fight. Now, I have changed, I got calmer here. • TP5 It helps mentally because it takes you out of the system in which you live, a system that is so absorbing. • TP1 I look into the mirror, and I am another person, not the same anymore.
		Possibility to express emotions (former prisoners)	• TP2 You could cry … you could play, be a child, be an angel. • TP5 It gives me a platform to investigate what I feel, the rage I feel. • TP3 Sometimes I want to cry and let it out. • TP4 I cried it all in the play. All that I could not cry at home, I cried in the theatre.
	Mental health problems	Control of addiction (former prisoners)	• TP1 Before, I was one of those who drank every week … drugs were also habitual. Now this changed me. Before, I wouldn’t even sit down at this table, because I was busy doing other things.
		Therapeutic effects (parents)	• TP4 And with the theatre, I could move on forward. They can never take away my pain, but I see things more calmly now. • TP3 If human beings keep all their emotions for themselves they hurt themselves. To the contrary here, you can let it all out, it feels good to cry. I cried it all here. • TP4 I need to go forward because here are children who suffer the same as my son suffered. • TP4 If I can help the guys who are imprisoned now, I will help them. • TP4 I like to see how the guys get the motivation to go ahead and move forward.
	Change of environment	New people	• TP2 Yes, I met many people and learned lots of things. • TP3 This has been crucial. As I told you, I could express myself, got to know more people, distinct experiences, things that would never have been at my reach.
		New places	• TP3 I would never have thought that I would be in a suit of a hotel. I had never travelled in airplane, boats, never (referring to invitations to theatre festivals). • TP4 Playing theatre helped me to get to know places that I would never have thought to get to know. • TP3 We feel liberation here—for example, in the settlement, where we live, exist lots of drugs; on Fridays there are fights and shootings everywhere; it feels like receiving a present to leave for a while.
	Interpersonal relationships	Positive aspects	• TP1 In everyday life, including the relationship with my family and with my close ones, I made them feel proud, pleased. • TP2 And now, I have people who help me, who support me, I have everything I need. It now is on me. • TP1 And I could even conquer a girl.
		Negative aspects	• TP3 The negative aspect is that I have another boy who is doing drugs unfortunately, and I am worrying to let him at home alone. The only negative aspect about doing theatre. • TP5 It starts to affect my time, and I have had less time with my daughter. This could be a negative aspect in parenthesis.
Possible mechanisms of change related to drama	Commitment with the drama production	Personal commitment and commitment with the group	• TP3 You cannot fail, if you fail you need to go on and work. • TP1 To not fail the others … to not fail yourself • TP4 Suddenly, you feel the urge to say: “I will not continue anymore,” but this does not work. One just has to face everything that comes, if you are in it … • TP1 You know, in this thing, it is not only you, but a group. If you fail, the group fails.
		Liking drama	• TP4 I got to know theatre only a short time ago, and I am in love with it. • TP5 Yes, because I like the theatre, I like it, I read a lot, I damn well like the theatre.
		Learning with drama	• TP4 And I now know to save things from the theatre. I know what happens, what they teach you, and I am learning. • TP1 But this time, it is different because here is practically a school. • TP4 The theatre requires you to pronounce things well, to speak well, to have a good presentation with other people. All this is education.
	Drama as an experience	Challenging rehearsals	• TP4 Suddenly, they criticize us because we are not doing things well. • TP1 You think you are performing well, and the director says: “No, do it again.” • TP3 Suddenly, I do the things right, and they tell me: “No, you have to do it this way and that and the other way” and I: “But I already did it.” Then, you have a setback.
		Identification with the roles	• TP3 The role I am playing in Richard III is a luxurious king, a pig, a bastard. So, it cost me, but I already mentalized it. • TP2 It is complex. There are texts which are mine. Things that I have lived that concern me. Lots. In fact, I created this role. • TP2 And suddenly the role beats me. Yes, it makes me be him a little bit in specific situations. It comes over me. • TP5 The person I enact is always furious, like an animal, angry, barking like a dog. Therefore, one lives this emotion, always angry, but when I leave the play, I totally change.
		Emotions associated with the acting	• TP4 All that crying I could not do at home, I did in the play/on stage. • TP5 When the text gets to me, I feel emotions. • TP1 Happiness. I went through many emotions, lots of anger and frustration.
		Feelings associated with the participation	• TP4 I feel free when I arrive here. • TP1 I like to be here more than anywhere else, even if I do not make a peso. • TP4 Yes, it is very nice, you can feel free from all problems. • TP5 It helps mentally to leave the system, in which you live, for a bit, which is a system that absorbs you.
	Relationships in the production	With other participants in rehabilitation	• TP1 And here they are, they are with you, helping you, and supporting you. • TP5 Then, you need to have positive energy. I get along well with the others. • TP4 Because you get to really know the others. They are modest, caring, you get to know another family. • TP5 Drama is a work in a group steered by the director. The actors create the roles together with the director, even the hairdresser, the illuminator, the guy who makes the sound, are in the group process.
		With the director	• TP3 She (the director) made me witness everything I had lived, what had happened. • TP2 I had problems and went to her deadly hurt. Hitting her door. She opened the door and told me: “Look, I have the following suggestion…” • TP 4 With her, we are together.
		With professional actors	• TP3 And you get to know new actors, and suddenly there is jealousy among the actors. • TP4 The actors, girls, who played in Torre 5, are here again, they are very nice.
	Drama as platform for social criticism	Criticism of censorship	• TP4 Here in Chile, everything is more closed, because they do not want the corruption in the prison system to become publicly known. They do not want that it becomes known that the prison guards are involved in the corruption. • TP5 I think they do not allow the company to enter prisons because of the conscience that is the fear of the prison administration, the government, the politicians. The prisons serve the politicians.
		Criticism of the national reality	• TP4 The things that happen in this country call attention, with all the people who are imprisoned, the corruption everywhere. • TP5 What happened if they would govern one day? Then, the country would have a destiny. We clearly say it to the politicians.
`		Criticism of the prison reality	• TP3 Because in this play, we destroy the prison guards. • TP4 Because you find everything in the play, they actually do in prison, you face the reality of what you do in prison.
		Creating conscience	• TP5 Exactly, it creates social conscience: social conscience, and when you are in prison, they come to tell you: “Look, you have to wake up; do not let them torture you.” • TP5 And maybe this generates fear in the prison administration that the theatre will wake up the prisoners, and they will begin to claim their rights. • TP2 This is your truth. It is like seeing things through a veil, like blurred. Getting at this state makes you see the reality, like cleaning the lens.
	Perceived necessity to play drama in prison contexts	As rehabilitation	• TP4 But with respect to rehabilitation, you would have to work with the guys inside prison. To get them closer. • TP4 And now with more reason, the play is with the boys to reinsert them into society, very nice.
		As treatment of mental health problems or promotion of mental health	• TP5 Good, totally good. It is health, the theatre does good to the mental health of prisoners. • TP5 This should be that they do not go out and kill each other … they kill each other for drugs.
		Need to have a policy	• TP5 Prison theatre should be implemented as policy, because it really helps a lot. • TP4 I think they should allow that the play is shown inside and the things that could be done for the prisoners. Show plays and produce plays with them inside.
	Future work perspectives related to theatre productions	Wish to continue in similar projects	• TP5 (more work) Yes, I think so … the director has planned more presentations. We will go on tour. • TP4 Maybe they do not only need the young people, and we could be in the crew again.
		Continuing to work related to theatre	• TP1 I would like to teach and try to return all what we could learn and experience, return, and try to teach as best as I can. • TP2 You do not know the people who will watch the play … but there could be directors, people who do castings, producers. Everything helps and would be welcome.
	Performing the play: transforming aspects of reaching the goal	Perception of the public and family	• TP3 There is a forum. Yes, a forum. A very good reception in Maipú (borough of Santiago); I was surprised because the spectators understood the play from the first minute to the last. • TP5 To have the applause, all this, when you finish the play, everybody comes and hugs; they congratulate you, it is awesome. • TP1 Then they suddenly recognize you, give you presents. This is like a nutrient, do not know whether this is ego, but it makes you feel that you are valued. • TP3 Now, the families were stunned. Many family members came up to me to congratulate me.
		Perception of the own achievement	• TP3 The good experience that I made with this production, unlike the anterior, when I was nervous, every time I went on stage, here I felt the contrary, totally relaxed. • TP 3 And thank god, everything went well. As I told you, I had the roles internalized. • TP5 They congratulate me that the play was beautiful—for example, they tell me it was brilliant. • TP1 And now, yes, I can, but not at the first attempt, the first attempt was as I thought it would go, but the second was better, and the third will be good.

**Figure 1:**
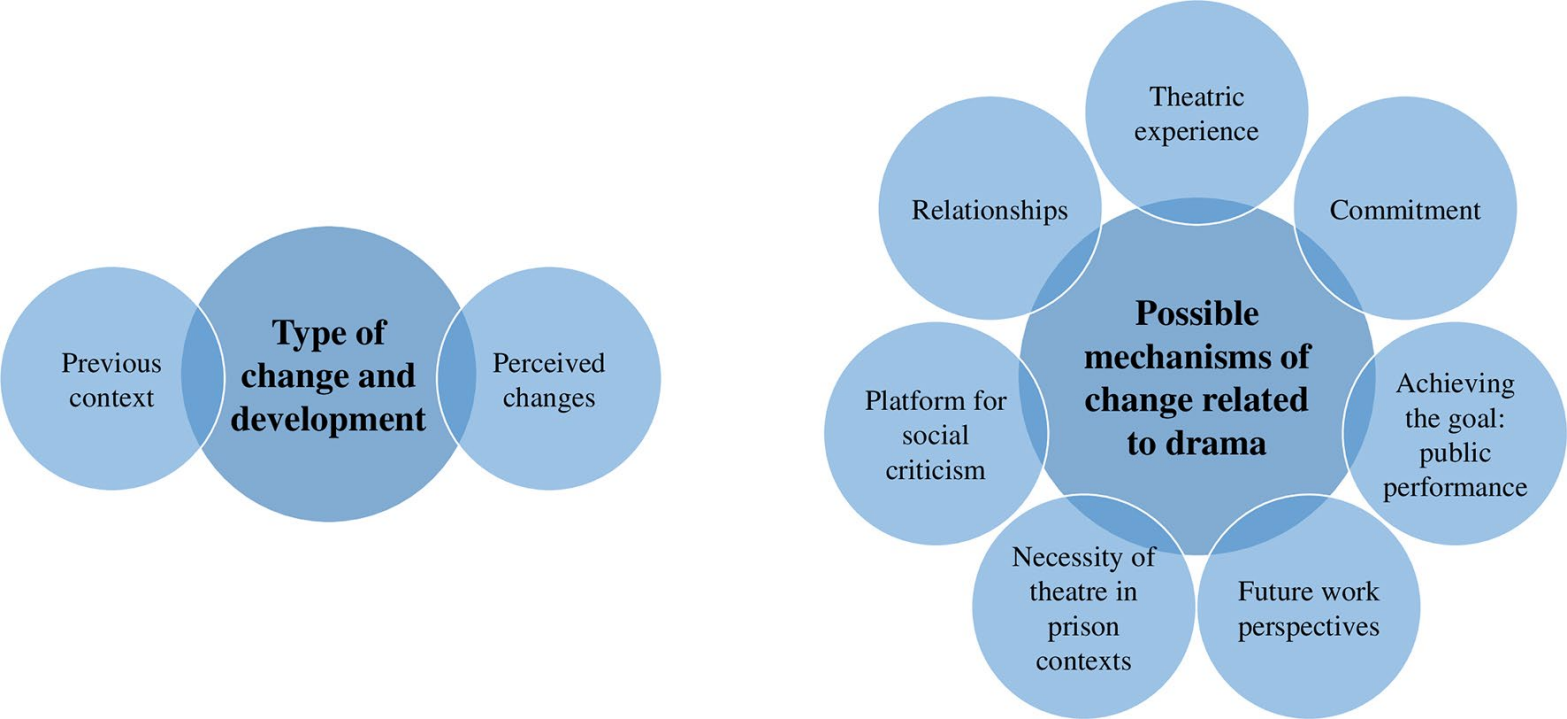
Type of change and possible mechanisms of change in individuals involved with the criminal justice system participating in a drama project.

**Figure 1** shows the two main themes, two subthemes for the first main theme, and seven categories for the second main theme.

### Types of Change and Development

This first theme includes the types of changes the participants perceived over the course of the intervention/participation in the theatre project. It includes descriptions of the previous context relating to mental health and substance use problems (personal characteristics, problems regulating interpersonal relationships, previous experiences in prison, traumatic experiences) and the perceived changes (with respect to mental health, substance use, social environment, affectivity, and personality).

Assessing the previous context, participants revealed personal characteristics that related to their mental health problems.

*TP5: “For example … I am very … was very impulsive … wherever I arrived, I immediately got into fights”*

When participants revealed previous states of mental health, the data were dominated by “substance abuse and addiction” especially for the former inmates.

*TP1: I was in a very bad place in this period of drugs, alcohol”*

The parents of the inmate who died in a prison fire participants expressed during the interviews emotions related to trauma and depression.

*TP3: “Five years have passed and it still hurts so much (crying)”*

All former inmates revealed negative experiences with prison life, perceived repression, and suffered the poor housing conditions in prison.

*“TP2: “Your skin is itching from the bedbugs and unbearably unhealthy. In a space … a little smaller than this room (about 2 x 3 meters) and it was like 1,80 or 1,90 meters by 2,10 meters were living 8 to 12 people. In a second floor inserted there were 3, 5 or 10 more…”*

The former inmates revealed emotions related to deprivation and the loss of relationships and material goods due to the substance use problems and imprisonment.

*“TP2: Sadness … I had money, I had a home, I had a wife, I had everything to be happy and I did not make it … because of my vices (refers to substance use).”*

The former inmates described their social context and interpersonal relations as obstacles for overcoming mental health issues.

*“TP1: I have a partner that has the same problem, you know? And we are about to break up, despite of all the love, all the tenderness we had because we potentiate each other when it comes to drugs”*

They described that mental health problems and substance use problems improved with participation in the project.

*“TP1: Before, I was one of those who drank every week … drugs were also habitual. Now this changed me. Before, I wouldn’t even sit down at this table because I was busy doing other things”*

“*TP4: And with the theatre I could move on forward. They can never take away my pain, but I see things more calmly now*”

### Possible Mechanisms of Change

The second theme describes possible mechanisms that underlie changes produced through the participation in theatre.

There was the possibility to express and reflect emotions related to traumatic experience.

*“TP4: All that crying, I could not do at home, I did in the play/on stage”*

The participants identified with the role and created new personal narratives.

*“TP2: It is complex. There are texts which are mine. Things that I have lived, that concern me. Lots. In fact, I have created this role.”*

The participation may have produced changes of self-perception affecting their identity:

*“TP1: I look into the mirror and I am another person, not the same anymore.”*

The positive reception of the play by the audience and their families was rewarding and changed the participants’ self-image. Participants expressed the experience of a personal success.

*“TP5: They congratulate me, that the play was beautiful, for example, they tell me it was brilliant”*

The change of the social environment was a recurrent theme. Participation in the theatre company meant the opportunity to meet new people and see new places.

*“TP3: This has been crucial. As I told you, I could express myself, got to know more people, distinct experiences, things that would never have been at my reach.”*

Changes in interpersonal relationships were described as positive.

*“TP1: In everyday life, including the relationship with my family and with my close ones, I made them feel proud, pleased”*

The group of former inmates emphasized the possibility to engage with people and to acquire new skills.

*“TP1: Theatre helped me to engage with people, and in being more disciplined”*

A crucial point for all was the commitment with the group to achieve the common goal of the project, to put the play on stage. The interpersonal relationships were perceived as resources during the participation in the theatre project.

*“TP5: I get a lot of support from my family … It consists of my little daughter … and my wife who also plays theatre; she is also an actress; so I have all their support”*

The relationships with the professional actors and the director played an important role for the participants going as far as referring to them as “family.” All participants had in common that they expected future activities or even remunerated jobs in theatre beyond the participation in the current project. Given that most of the participants had previously participated in a prison theatre project that had been successful with a series of invitations to international festivals, the aspiration did not seem to be completely impossible, but unrealistic.

Participants also described theatre as a platform for social criticism that would not be heard elsewhere. The play reached general public interested in experimental and alternative theatre. It was also discussed in the media. The opportunity to criticize authorities, police, censorship, and politics in front of a public outside, and beyond their usual social environment generated a feeling of satisfaction. Shakespeare seemed to be a safe mechanism to serve this purpose through his cultural power but historical distance. Theatre was described as a way of generating more awareness about imprisonment, inside and outside prison. Participants expressed the idea that more theatres inside the prisons would be useful for rehabilitation, social reinsertion, and improvements of mental health.

*“TP5: Exactly, social awareness is generated: A social awareness and when you are a prisoner it tells you ‘Look, you have to wake up, don’t let the guards torture you.’”*

For two of the former inmates as well as the mother of the inmate who died in the prison fire, the artistic work was a vehicle of change superior to psychotherapeutic treatments and medication. They reflected on their past contacts with psychiatrists, therapists, and treatments that had not been useful to them.

*“TP1: I don’t like therapies and I don’t like pills”*

### Quantitative Content Analysis

The number of appearance of quotes and codes per category and theme, and by how many participants each category and theme was brought up, are reported in **Table 3**.

**Table 3:** Quantitative content analysis indicating the number of codes and number of quotes per category and theme, and by how many participants each category, and theme was brought up.

	Number of codes	Number of quotes	Number of participants (N = 5)
**Theme: types of change and development**			
Total in theme	25	70	5
**Category**			
**Subtheme: previous context**	17	46	5
Personal characteristics	2	6	4
Mental health problems	2	5	5
Personal reflections	2	6	3
Interpersonal relationships	2	5	3
Prison experience	3	7	3
Motivation to participate in drama	6	17	5
**Subtheme: perceived changes**	8	24	5
Personal level	2	8	5
Mental health problems	2	6	3
Change of environment	2	5	3
Interpersonal relationships	2	5	4
**Theme: possible mechanisms of change related to drama**			
Total in theme	21	59	5
**Category**			
Commitment with the drama production	3	9	4
Drama as an experience	4	14	5
Relationships during the production	3	9	5
Drama as a platform for social critique	4	9	4
Perceived necessity to play drama in prison contexts	3	6	2
Future work perspectives related to theatre productions	2	4	4
Performing the play: transforming aspects of reaching the goal	2	8	3

## Discussion

### Main Findings

Participation in theatre projects may subjectively change emotional states related to traumatic experiences and improve capacities to regulate interpersonal relationships in people with criminal justice involvement. The possible mechanisms include commitments with the group and with performing the work, expression of emotions, and traumatic experiences, being heard in public and positive perception by others. New skills and artistic success may open up personal perspectives and future projections.

### Strengths and Limitations

This was an innovative collaboration between professionals in drama with backgrounds in academia and in practice working together with professionals in mental health with backgrounds in psychology and in psychiatry to address research questions relating to drama interventions in a Latin American context. The participants had a similar socio-economic background that allows focusing on the effects of theatre on mental health and psychosocial functioning.

The study also had several limitations: the number of participants was small. The group of participants was heterogeneous. There were former inmates and the parents of an inmate who died in a prison fire. Participants had different types of traumatic experiences and criminal justice involvement. Not all the participants were available for a second interview after the performances. The small number of participants may not have yielded saturation of the themes and topics identified in the study. The results may have been influenced by the selection of participants with positive previous experiences of performing drama.

### Comparison With the Literature

There are two different aspects of using arts as therapeutic vehicles: 1) in the context of art therapies that focus on the rehabilitation process in workshops or rehearsals and 2) doing art with a focus on the artistic product that is exhibited or performed to communicate with the public, society, and the contemporary art scene.

The approach used in this study involves both aspects, which is potentially powerful. It stands in a South American landscape that includes such figures as Brazilian psychiatrist Nise da Silveira (46) who used arts as an access to the unconscious in psychotherapies and also focused on the artistic product facilitating success of several artists with mental health problems in the contemporary art scene. Beyond using arts in therapies, Silveira converted parts of a psychiatric hospital into an open creative laboratory that was used by patients and attracted the interest of critics, artists, and the general public intrigued and inspired by the art of the mentally ill. The hospital in Rio de Janeiro where she worked from the 1940s through to the 1990s now hosts the *Museum of Images of the Unconscious* which houses one of the world’s most important art collections produced by patients with mental illnesses. Formerly known as the Pedro II Psychiatric Centre, it has been renamed as the Nise da Silveira Municipal Institute.

Participating in theatre may improve personal development and rehabilitation of people with criminal justice involvement (47). In the treatment of traumatic stress, performing theatre was shown to lead to more self-awareness and positive psychological transformation (48). Elements of testimonial theatre or Boal’s concept of forum theatre encourage a simultaneous dramaturgy between actors and spectators. This was later refined to create a methodology Boal referred to as *The Rainbow of Desire* whereby participants introduce narratives of their personal lives and experiences in performing and understanding their own behaviors (13, 14). Drama therapy has been shown to enhance alternative behavioral choices that are crucial for achieving abstinence in the treatment of substance use disorders (49, 50). Performing *Richard III* implies identification with the roles and provides the opportunity to express traumatic experiences such as exposure to violence, which is common in prisoners (51). The performance of a role and the use of Shakespeare’s language allow the participants to communicate in ways that are different from their habitual form of interaction (33, 52).

All participants felt that they expressed social criticism by performing theatre, which is a crucial element of testimony therapy (28). Several participants of this study expressed the necessity of implementing theatre inside prisons not only to create social awareness inside prisons but also to communicate through this medium with a wider public. They experienced the possibility to criticize social inequalities, prison life, and the feeling of repression from police, politics, and authorities. In a previous theatrical production documenting a prison fire—in which most of the actors had performed, and several of the participants had lost family members—the criticism of the authorities had been more direct. They experienced opportunities to create awareness in society for the problems of marginalized communities and especially prison inmates. The role of theatre in prisons has been described as a social process *Staging Human Rights* (53). Prison theatre may be less effective when it merely focuses on the process of rehearsals (54). This study revealed that being heard and appreciated in public led to the experience of social inclusion and empowerment as possible mechanisms of mental health improvements in the participants. In the success of putting the play on stage, this individual and collective accomplishment contributes to positive emotions (55). Public success of performing prison theatre enables the actors to visualize an alternative self and gain motivation for their future (56).

### Conclusions

The participation in theatre productions seems to subjectively initiate change regarding the regulation of emotions and interpersonal relationships in people with criminal justice involvement. The mechanisms may go beyond and complement those of individual psychotherapies and medications. They include identification with roles, artistic expression, commitment with group processes, communication with the public, and social criticism. Wider scale research can be recommended on the type and size of these effects in larger and more homogeneous samples of participants. Contact procedures for follow-ups should be improved to reduce attrition in future research.

## Data Availability Statement

The datasets generated for this study are available on request to the corresponding authors.

## Ethics Statement

This study was carried out in accordance with the recommendations of “research ethics review board of the Universidad San Sebastián, Chile” with written informed consent from all subjects. All subjects gave written informed consent in accordance with the Declaration of Helsinki. The protocol was approved by the “research ethics review board of the Universidad San Sebastián, Chile.”

## Author Contributions

AM, JR, and PH contributed to the conception and design of the study. AM, PM, and CG collected the data. CG and CS performed the qualitative data analysis. AM and CG wrote the first draft of the manuscript. All authors critically revised the manuscript, and approved the submitted version. AM supervised the study.

## Funding

The study was supported by funding of the Universidad San Sebastián, Chile and by a bridging fund for exploring interdisciplinary collaborations in life sciences of the Wellcome Trust, UK. APM receives funding of the CONICYT, grant scheme FONDECYT Regular 1190613. We acknowledge support from the German Research Foundation (DFG) and the Open Access Publication Fund of Charité – Universitätsmedizin Berlin.

## Conflict of Interest

The authors declare that the research was conducted in the absence of any commercial or financial relationships that could be construed as a potential conflict of interest.
