# Corrigendum: Initiating Change of People With Criminal Justice Involvement Through Participation in a Drama Project: An Exploratory Study

**DOI:** 10.3389/fpsyt.2020.00139

**Published:** 2020-02-26

**Authors:** Adrian P. Mundt, Pamela Marín, Caroline Gabrysch, Carolina Sepúlveda, Jacqueline Roumeau, Paul Heritage

**Affiliations:** ^1^Medical Faculty, Universidad San Sebastián, Puerto Montt, Chile; ^2^Medical Faculty, Universidad Diego Portales, Santiago, Chile; ^3^Department of Psychiatry and Psychotherapy, Charité Campus Mitte, Universitätsmedizin Berlin, Berlin, Germany; ^4^Department of Psychiatry and Mental Health, Hospital Clínico Universidad de Chile, Santiago, Chile; ^5^Department of Psychology, Universidad del Humanismo Cristiano, Santiago, Chile; ^6^Corporación de Artistas por la Rehabilitación y Reinserción Social a través del Arte CoArtRe, Santiago, Chile; ^7^Department of Drama, Queen Mary University of London, London, United Kingdom

**Keywords:** drama, art therapy, prison population, mental disorders, addiction, substance use disorders

In the published article it should have been indicated that Caroline Gabrysch was a corresponding author.

The authors apologize for this error and state that this does not change the scientific conclusions of the article in any way. The original article has been updated.

